# *TP53* mutant cell lines selected for resistance to MDM2 inhibitors retain growth inhibition by MAPK pathway inhibitors but a reduced apoptotic response

**DOI:** 10.1186/s12935-019-0768-3

**Published:** 2019-03-07

**Authors:** Chiao-En Wu, Tsin Shue Koay, Yi-Hsuan Ho, Penny Lovat, John Lunec

**Affiliations:** 1 Northern Institute for Cancer Research, School of Medicine, Newcastle University, Paul O’Gorman Building, Medical School, Framlington Place, Newcastle upon Tyne, NE2 4HH UK; 2 Division of Hematology-Oncology, Department of Internal Medicine, Chang Gung Memorial Hospital at Linkou, Chang Gung University College of Medicine, Taoyuan, Taiwan; 3 Dermatological Sciences, Institute of Cellular Medicine, Newcastle University, Newcastle, UK

**Keywords:** Melanoma, p53, MDM2, Nutlin-3, RG7388, HDM201, Trametinib, Vemurafenib

## Abstract

**Background:**

Emergence of resistance to molecular targeted therapy constitutes a limitation to clinical benefits in cancer treatment. Cross-resistance commonly happens with chemotherapeutic agents but might not with targeted agents.

**Methods:**

In the current study, *TP53* wild-type cell lines with druggable MAPK pathway mutations [*BRAF*^V600E^ (WM35) or *NRAS* ^Q61K^ (SJSA-1)] were compared with their *TP53* mutant sublines (WM35-R, SN40R2) derived by selection for resistance to MDM2/p53 binding antagonists.

**Results:**

The continued presence of the druggable MAPK pathway targets in the *TP53* mutant (*TP53*^MUT^) WM35-R and SN40R2 cells was confirmed. Trametinib and vemurafenib were tested on the paired WM35/WM35-R and SJSA-1/SN40R2 cells and similar growth inhibitory effects on the paired cell lines was observed. However, apoptotic responses to trametinib and vemurafenib were greater in WM35 than WM35-R, evidenced by FACS analysis and caspase 3/7 activity, indicating that these MAPK inhibitors acted on the cells partially through p53-regulated pathways. SiRNA mediated p53 knockdown in WM35 replicated the same pattern of response to trametinib and vemurafenib as seen in WM35-R, confirming that p53 plays a role in trametinib and vemurafenib induced apoptosis. In contrast, these differences in apoptotic response between WM35 and WM35-R were not seen with the SJSA-1/SN40R2 cell line pair. This is likely due to p53 suppression by overexpressed MDM2 in SJSA-1.

**Conclusion:**

The TP53^MUT^ cells selected by resistance to MDM2 inhibitors nevertheless retained growth inhibitory but not apoptotic response to MAPK pathway inhibitors.

**Electronic supplementary material:**

The online version of this article (10.1186/s12935-019-0768-3) contains supplementary material, which is available to authorized users.

## Background

*RAS* and *RAF* oncogenes are frequently mutated in human cancer leading to a constitutively activated MAPK pathway which is critical for oncogenesis, tumour proliferation and survival [1, 2]. These genetic alterations provide important targets druggable by low molecular weight compounds which have been evaluated in clinical trials and become licensed treatments. For example, the dual blockade of the MAPK pathway by the combination of BRAF and MEK inhibitors has become the standard treatment for unresectable or metastatic melanoma harboring a *BRAF*^V600^ mutation [3, 4]. Vemurafenib [5] and trametinib [6] are the first BRAF and MEK inhibitors respectively to be approved for BRAF-mutated cancer (melanoma). Trametinib strongly inhibits MEK1/2 kinase activities and was shown not to inhibit another 98 kinase activities [7]. Preclinical study of trametinib showed efficacy against cancer cells with either *BRAF* or *RAS* mutations and even on cancer cells with neither *BRAF* nor *RAS* mutations [8]; therefore, cancers without *BRAF* mutations are included in clinical trials of trametinib.

Tumour protein p53, encoded by the tumour suppressor gene *TP53*, is a transcription factor activating target genes that mediate various functions, including deoxyribonucleic acid (DNA) repair, metabolism, cell cycle arrest, apoptosis, senescence and autophagy [9–11]. Loss of wild-type (WT) p53 function by either *TP53* mutations, or overexpression of its negative regulators such as MDM2, causes cancer cell development, survival, and proliferation [12]. MDM2-p53 binding antagonists are designed to occupy the p53-binding pocket of MDM2 and therefore stabilize p53 through prevention of MDM2-mediated ubiquitylation and proteasomal degradation. MDM2-p53 binding antagonists cause cell cycle arrest, apoptosis, and growth inhibition of cancer cells resulting from activation of the p53 pathway in p53^WT^ (p53-wild type) cancer cells [13]. The first small molecule MDM2 inhibitor, Nutlin-3, showed efficacy in vitro and with tumour xenograft (SJSA-1) models in nude mice [14]. RG7388 [15] and HDM201 [16] are new generations of MDM2 inhibitors which are more potent and specific than Nutlin-3 and clinical trials are ongoing to investigate their efficacy in clinical settings.

Emergence of resistance to molecular targeted therapy constitutes a limitation to maintained clinical benefits in cancer treatment. Cross-resistance commonly happens with chemotherapeutic agents. After selection for resistance to a single drug, cells frequently also show cross-resistance to other structurally and mechanistically unrelated drugs by increasing the activity of efflux pumps (*p*-glycoprotein, PGP, also known as multidrug resistance protein 1, MDR-1; multidrug-resistance-associated protein 1, MRP-1) or reducing drug influx, or other mechanisms [17].

Unlike cytotoxic agents, resistance to targeted therapy usually results from mutation of the target gene or activation of pro-survival signaling pathways [18]. Because of the different resistance mechanisms involved, cross-resistance might play less of a role in targeted therapy than with cytotoxic agents. The evidence for cross-resistance of targeted therapies, particularly for MDM2 inhibitors is lacking. Michaelis et al. selected p53 mutant (*P53*^MUT^) cells (UKF-NB-3^r^ Nutlin^10μM^) by continuous exposure to Nutlin-3 and found the resistant cells displayed a multi-drug resistant phenotype which was resistant to various cytotoxic drugs and irradiation [19]. By contrast, another study showed the resistant cells (S_N40R2, N_N20R1) selected by Nutlin-3 retained sensitivity to ionizing radiation [20]. Previous studies have focused on the cross-resistance between MDM2 inhibitors and cytotoxic agents and irradiation, but to date no reported studies have investigated the response to alternative effective small molecule targeted agents in cells selected for resistance to MDM2 inhibitors.

The aim of the current study was to select cell lines with resistance to MDM2/p53 binding antagonists from cancer cells with druggable targets of the MAPK pathway resulting from either *BRAF*^V600E^ (WM35) or N*RAS*^Q61K^ (SJSA-1) mutation and to examine whether the MDM2 inhibitor-resistant cell lines retain sensitivity to the MAPK pathway inhibitors, vemurafenib and trametinib.

## Methods

### Cell lines and reagents

Two parental and MDM2 inhibitor resistant cell line pairs, WM35/WM35-R and SJSA-1/SN40R2, were routinely cultured using Dulbecco’s modified Eagle’s medium (DMEM) medium and RPMI-1640 medium (Sigma, Dorset, UK) respectively, which were supplemented with 10% (v/v) foetal calf serum. All the cell lines were authenticated by serial tandem repeat (STR) profiling (WM35, WM35-R by NewGene, Newcastle, UK; SJSA-1, SN40R2 by LGC Standards). Nutlin-3 was purchased from NewChem (Newcastle, UK), RG7388 and HDM201 were obtained by custom synthesis via Astex Pharmaceuticals. Trametinib and vemurafenib were obtained from Cambridge Bioscience. All compounds were initially dissolved in dimethyl sulfoxide (DMSO) (Sigma-Aldrich) and used to dose cells at a final concentration of 0.5% DMSO, optimised to give minimal cytotoxic effects on cells, and 0.5% DMSO only solvent controls were included in all experiments.

### Growth inhibition assay

Cells were seeded in 96-well plates overnight and treated with indicated drugs for 72 h. The cells were fixed using Carnoy’s fixative followed by Sulforhodamine B (SRB) assay [21]. A spectrophotometer (Spectramax250 Molecular Devices) was used to measure absorbance at 570 nm. The GI_50_ value was determined by the concentration of a compound which can reduce the growth of the cell population by 50% compared to untreated control cultures after subtraction of baseline seeded cell amount prior to the start of treatment [22].

### Immunoblotting

Cells lysates were harvested by scraping and suspension in lysis buffer (62.5 mM Tris HCl/pH 6.8, 10% glycerol and 2% SDS), heated and sonicated. The protein concentrations of the cell lysates were estimated using a Pierce^®^ BCA Protein Assay kit. Equal quantities of protein were loaded onto and separated by SDS–polyacrylamide gels (4–20% Mini-PROTEAN^®^ TGX™ Gel, BioRad). The separated proteins were transferred and immobilized onto Amersham™ nitrocellulose membranes (GE Healthcare Life Science). Primary antibodies against p53 (DO-7) (#M7001, Dako), MDM2 (Ab-1) (#OP46, Merck Millipore), p21^WAF1^ (EA10) (#OP64, Calbiochem), p-ERK (E-4) (sc-7383, Santa Cruz), ERK (K-23) (sc-94, Santa Cruz), GAPDH (14C10) (#2118, Cell Signaling Technology), BRAF^V600E^ (VE1, Spring Bioscience), Actin (A4700, Sigma) and secondary goat anti-mouse/rabbit horseradish peroxidase-conjugated antibodies (#P0447/P0448, Dako) were used. All antibodies were diluted in 5% (w/v) non-fat milk or BSA in TBS-tween (20 mM Tris/pH 6.8, 137 mM NaCl, 0.1% tween-20). Protein signals were visualized using enhanced chemiluminescence (GE Healthcare Life Sciences) and X-ray film (Fujifilm).

### RNA extraction and qRT-PCR (quantitative real-time polymerase chain reaction)

Total RNA was extracted using an RNeasy Mini Kit (Qiagen, Germany). RNA purity and concentration were estimated with an ND-1000 spectrophotometer (NanoDrop Technologies, Thermo Scientific, UK). Complementary DNA was generated using the High-Capacity cDNA Reverse Transcription Kit (4368814, Thermo Fisher Scientific) as described by the manufacturer. qRT-PCR was carried out using SYBR green RT-PCR master mix (Life Technologies) as per the manufacturer’s guidelines and the following primers:

- *MDM2*: F-AGTAGCAGTGAATCTACAGGGA, R-CTGATCCAACCAATCACCTGAAT
- *CDKN1A*: F-TGTCCGTCAGAACCCATGC, R-AAAGTCGAAGTTCCATCGCTC
- *PUMA*: F-ACCTCAACGCACAGTACGA, R-CTGGGTAAGGGCAGGAGTC
- *BAX*: F-CCCGAGAGGTCTTTTTCCGAG, R-CCAGCCCATGATGGTTCTGAT
- *PIG*-*3*: F-AGCGAGGAAGTCTGATCACC, R-CGTGGAGAAGTGAGGCAGAA
- *AEN*: F-CTTCCAGGCGCTCAAGTATGT, R-GGGCCAGGTCCTTTAGAGAGA
- *FDXR*: F-CAGCATTGGGTATAAGAGCCG, R-GGCCTGGCACATCCATAACC
- *TNFRSF10B*: F-ATGGAACAACGGGGACAGAAC, R-CTGCTGGGGAGCTAGGTCT
- *TP53INP1*: F-TCTTGAGTGCTTGGCTGATACA, R-GGTGGGGTGATAAACCAGCTC
- *TP53:* F-CAGCACATGACGGAGGTTGT, R-TCATCCAAATACTCCACACGC
- *GAPDH*: F-CAATGACCCCTTCATTGACC, R-GATCTCGCTCCTGGAAGAT.

A total of 10 ng of the cDNA samples per 10 µL final reaction volume, with the standard cycling parameters (stage 1: 50 °C for 2 min, stage 2: 95 °C for 10 min, then 40 cycles of 95 °C for 15 s, and 60 °C for 1 min), were set and carried out on an ABI 7900HT sequence detection system. GAPDH was used as endogenous control and samples of cells exposed to DMSO carrier were used as the calibrator for each independent repeat, with the formula 2^ΔΔCt^ used to calculate fold-changes. Analysis was carried out using SDS 2.3 software (Applied Biosystems).

### Fluorescence-activated cell sorting (FACS)

After treatment, floating and adhered cells were pooled and fixed using 70% cold ethanol. Samples were incubated in 250 μL PBS with 40 μg/mL propidium iodide (Sigma-Aldrich), 20 μg/mL RNAse A (Sigma-Aldrich) for 20 min in the dark at room temperature, then were analyzed on a FACSCaliburTM flow cytometer using CellQuest Pro software (Becton–Dickinson, Oxford, UK). Cell cycle distribution based on DNA content was determined using Cyflogic (CyFlo Ltd, Turku, Finland).

### Caspase 3/7 activity assay

Melanoma cells were seeded in white 96-well plates and treated after 24 h. Caspase-3/7 enzymatic activities were measured using a FLUOstar Omega plate reader (BMG Labtech) after adding a 1:1 ratio of CaspaseGlo 3/7 reagent (Promega) to growth media and incubating for 30 min. All values were expressed as a ratio of signal relative to solvent control.

### DNA sequencing and mutation-specific PCR

Sanger sequencing for WM35-R was commercially provided by NewGene. Total DNA was extracted using a DNeasy Blood & Tissue Kit (QIAGEN Inc., UK) and PCR used to amplify the indicated genes. PCR products were purified using a QIAquick PCR purification kit (QIAGEN Inc., UK) and sequenced by PCR based Sanger dideoxy chain termination.

- *NRAS:* F-5′-CCACACCCCCAGGATTCTTAC-3′, R-5′-AGTGTGGTAACCTCATTTCCC-3′
- *TP53*(exon10): F-5′-CATGTTGCTTTTGTACCGTCA-3, R-5′-TGAAGGCAGGATGAGAATGGA-3′.

Mutations detected by sequencing were then investigated by mutation specific PCR.

Primer pairs specific to either wild-type or mutant *TP53* were designed as follows:

- Forward wild type: 5′-CTGTTGCTGCAGATCCGTGG-3′,
- Forward mutant: 5′-CTGTTGCTGCAGATCCGTGT-3′,
- Reverse: 5′-CCTTTGACCATGAAGGCAGGA-3′.

Following PCR, products were analysed on 2% agarose gels containing ethidium bromide and were visualised by UV light (G:BOX imaging system).

### siRNAs and transfection

40 nM siRNA duplex against p53 was transfected by Liofectamine 2000 (Thermo Fisher Scientific) in OptiMEM-glutamax (Optimem) serum free media (Invitrogen). The sequences were designed as following:

- SiRNA of p53 (SiP53)
- Sence 5′-CCACCAUCCACUACAACUAdTdT-3′
- Antisence: 5′-UAGUUGUAGUGGAUGGUGGdTdT-3′
- SiRNA of control (SiControl)
- Sense: 5′-GCGCGCUUUGUAGGAUUCGdTdT-3′
- Antisense: 5′-CGAAUCCUACAAAGCGCGCdTdT-3′.

### Statistical analysis

Data were presented as mean ± standard error of mean (SEM) unless otherwise stated. Statistical tests were carried out using GraphPad Prism 6 software and all p-values represent paired t-tests of at least three independent repeats. A p-value less than 0.05 was considered as statistically significant.

## Results

### Selection for resistant cells

SN40R2 and WM35-R cell lines with resistance to MDM2/p53 binding antagonists were generated by continuously exposing SJSA-1 osteosarcoma cells [20] and WM35 cutaneous melanoma cells to either Nutlin-3 or RG7388 respectively. WM35 parental cells were cultured in medium with 0.5 μM RG7388 in 175 cm-squared flasks and then RG7388 was escalated to 1, 2, 3, 5 μM gradually within 3 months. Paired WM35/WM35-R and SJSA-1/SN40R2 have BRAF^V600E^ and NRAS^Q61K^ mutations which activate the MAPK pathway and render the cells druggable by MEK or BRAF inhibitors.

### WM35-R cells are resistant to other MDM2 inhibitors

**Fig. 1:**
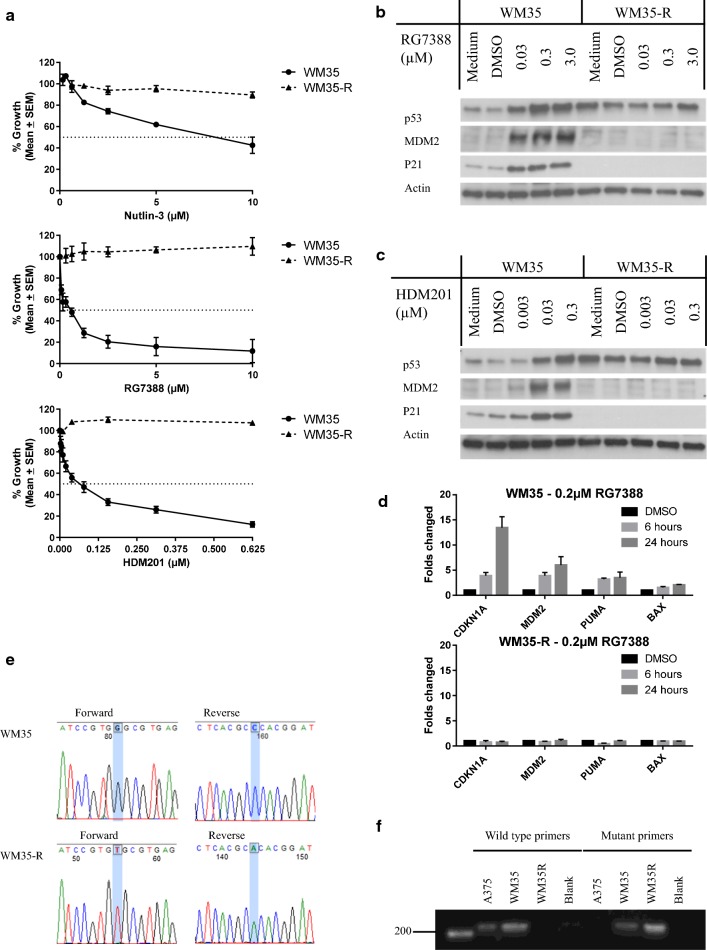
WM35-R was selected from WM35 for resistance to MDM2 inhibitors. **a** Growth inhibition curves for the response of WM35 and WM35-R cells to treatment with MDM2 inhibitors for 72 h. Immunobloting of WM35 and WM35-R following treatment with MDM2 inhibitors, RG7388 (**b**) and HDM201 (**c**) for 6 h. **d** *CDKN1A*, *MDM2*, *PUMA* and *BAX* expressions by qRT-PCR in WM35 and WM35-R following treatment with 0.2 µM RG7388 for 6 and 24 h. **e** Chromatogram showing p53 mutations in WM35-R. **f** Mutation specific PCR detecting the presence of a low frequency of mutant alleles in the parental WM35 cell population

WM35-R cells were selected from a parental WM35 culture by exposure to a final concentration of 5 μM RG7388. The WM35-R selected cells were treated with a range of RG7388 doses for 72 h in 96 well plates followed by SRB growth inhibition assay to confirm its resistance. The resistant cells underwent STR profiling to confirm that the selected daughter cells were derived from their parental cell lines (Additional file 1: Figure S1). To evaluate whether WM35-R cells were cross-resistant to other MDM2 inhibitors, WM35-R cells were treated with Nutlin-3, RG7388, and HDM201 for 72 h. The growth inhibition curves confirmed WM35-R was cross-resistant to other MDM2 inhibitors (Fig. 1a). To understand the mechanism of resistance to MDM2 inhibitors, immunoblotting and qRT-PCR were performed. Immunoblotting showed p53 stabilization, followed by inductions of MDM2 and p21 after RG7388 and HDM201 treatment in WM35 but not in WM35-R, indicating functional inactivation of p53 in WM35-R (Fig. 1b, c). Further investigation of transcripts for p53-targeted genes by qRT-PCR confirmed lack of induction for *CDKN1A, MDM2, PUMA, BAX*, in WM35-R after 6- and 24-h RG7388 treatment (Fig. 1d). To identify the most likely mechanism of resistance to MDM2 inhibitors, Sanger sequencing of *TP53* was carried out for WM35-R and revealed a homozygous *TP53* point mutation (c.1001G>T) resulting in a p.Gly334 Val amino acid substitution in the p53 tetramerization domain, which was not found in the parental WM35 cells (Fig. 1e). To examine whether parental WM35 cells already harbour p53^G334V^ mutant subclones, which were not detectable by Sanger sequencing or functional assays, mutation-specific PCR was performed. Primers were designed specific for this point mutation and revealed that the parental WM35 cell population harboured a low frequency of this *TP53* mutant allele (Fig. 1f). No evidence of the WT *TP53* allele was found by sequence-specific PCR in the selected WM35-R cell population. A375, a *TP53* wild-type melanoma cell used as a negative control, showed no evidence of bands specific for this *TP53* mutation. Therefore, it was concluded that a subpopulation of cells with resistance to MDM2 inhibitors, harbouring p53^G334V^ mutations, was present in the predominantly p53^WT^ WM35 parental cell culture and was selected by continuous culture in medium with RG7388.

### SN40R2 cells selected from SJSA-1 are resistant to all MDM2 inhibitors tested

**Fig. 2:**
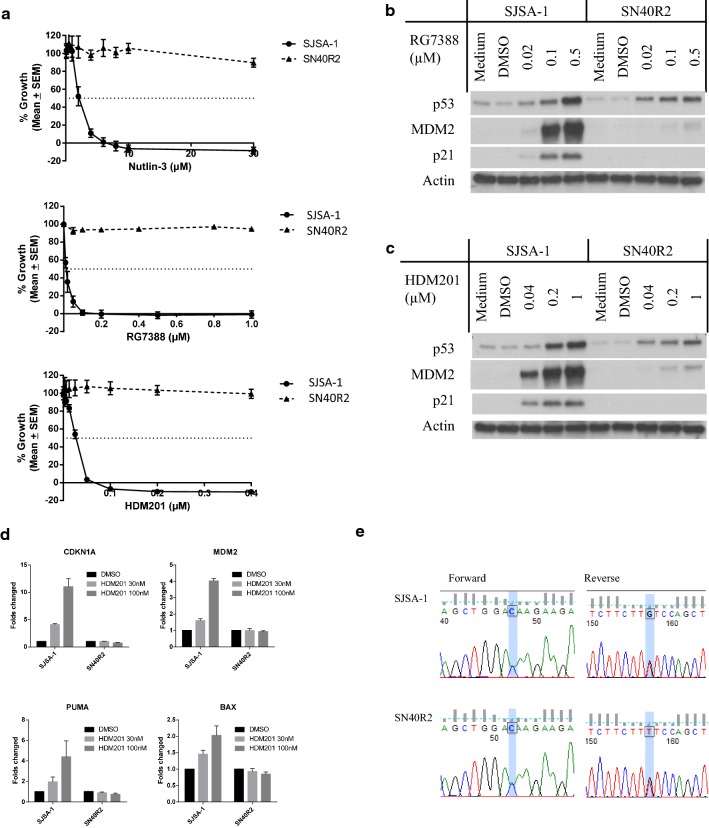
SN40R2 selected from SJSA-1 was resistant to MDM2 inhibitors and retained an *NRAS* mutation. **a** Growth inhibition curves of SJSA-1 and SN40R2 treated with MDM2 inhibitors for 72 h.** b**,** c** Immunobloting of SJSA-1 and SN40R2 treated with MDM2 inhibitors, RG7388 (**a**) and HDM201 (**c**) for 6 h. **d** *CDKN1A*, *MDM2*, *BBC3*(*PUMA*) and *BAX* expressions by qRT-PCR in SJSA-1 and SN40R2 treated with HDM201 for 6 h. **e** Chromatogram showing a c.181C>A *NRAS* mutation in both SJSA-1 and SN40R2

SN40R2 cells were selected from a parental SJSA-1 culture following exposure to a final concentration of 40 μM Nutlin-3. SN40R2 was authenticated and confirmed by STR profiling to be derived from SJSA-1. *TP53* mutations were found in codon 285 in SN40R2 [20]. Cross-resistance to MDM2 inhibitors, Nutlin-3, RG7388 and HDM201, was also found for SN40R2 (Fig. 2a). Western blotting showed dose-dependent increases of p53 and MDM2 after treatments of RG7388 and HDM201 in both cells. However, MDM2 expression in SN40R2 was much lower than SJSA-1 and p21 induction was evident in SJSA-1, but not SN40R2 (Fig. 2b, c). MDM2 mRNA was not induced after HDM201 treatment indicating that the increase of MDM2 protein in SN40R2 resulted from stabilization by MDM2 inhibitors rather than transactivation by p53 stabilization (Fig. 2d). The SJSA-1 cell line was known to harbour an *NRAS* mutation. To confirm retention of the *NRAS* mutation, a potential druggable target for MEK inhibitors, sanger sequencing was performed and showed that both SJSA-1 and SN40R2 harboured a heterozygous *NRAS* point mutation (c.181C>A) resulting in a p.Gln61Lys amino acid substitution in the NRAS protein (Fig. 2e).

### Growth inhibition of paired WM35 and WM35-R cells by trametinib and vemurafenib

**Fig. 3:**
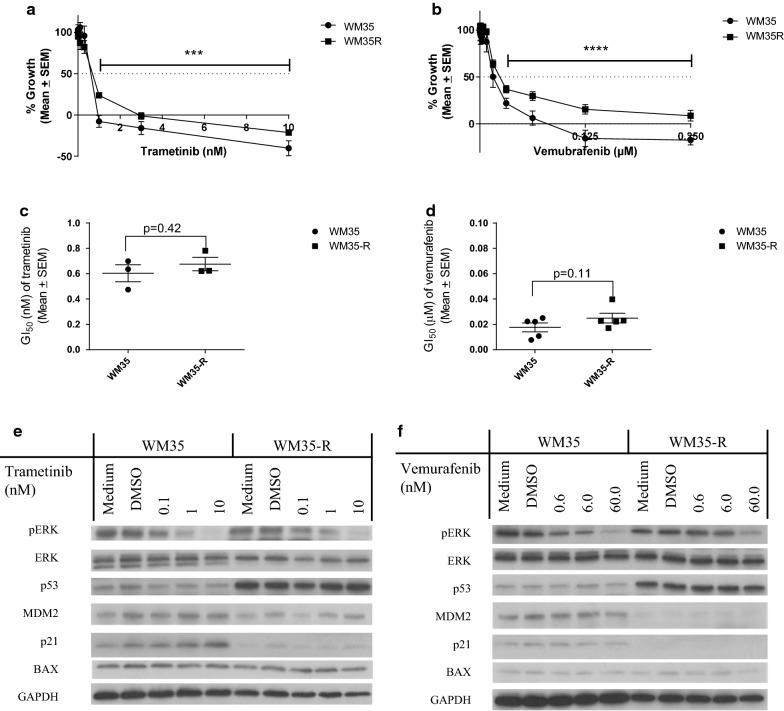
WM35 and WM35-R were treated with trametinib or vemurafenib. Growth inhibition curves by SRB assay for WM35 and WM35-R treated with trametinib (**a**) or vemurafenib (**b**) for 72 h. Two way ANOVA was used to test the difference between the cell lines for concentrations higher than GI_50_ (***p < 0.001; ****p < 0.0001). Comparison of GI_50_ for trametinib (**c**) and vemurafenib (**d**) for WM35 and WM35-R. Immunobloting of WM35 and WM35-R following treatment with trametinib (**e**) or vemurafenib (**f**) for 6 h

Because the paired WM35 and WM35-R harbour druggable BRAF^V600E^ mutations (confirmed by BRAF^V600E^ specific antibody, Additional file 1: Figure S2) their responses to the MAPK pathway inhibitors vemurafenib and trametinib were compared. Growth inhibition by SRB assay showed the parental WM35 cells were slightly but non-significantly more sensitive, in terms of GI_50_ level, to either trametinib or vemurafenib (Fig. 3a–d). However, the growth inhibition curves plotted with Day 0 subtraction went further below the seeding population level (X axis) for WM35 compared with WM35-R, suggesting more cell killing and not just growth inhibition was induced in WM35 than WM35-R after trametinib and vemurafenib treatment. A significant difference was found when the growth inhibitory curves above GI_50_ were compared by 2 way ANOVA (p = 0.0006 for trametinib and p < 0.0001 for vemurafenib). Immunoblotting showed dose dependent suppression of phospho-ERK by 6-h treatment with trametinib or vemurafenib for both cell lines, confirming inhibition of MAPK pathway activity (Fig. 3e, f).

### The effect of trametinib or vemurafenib treatment on cell cycle distribution and apoptosis for paired WM35 and WM35-R

**Fig. 4:**
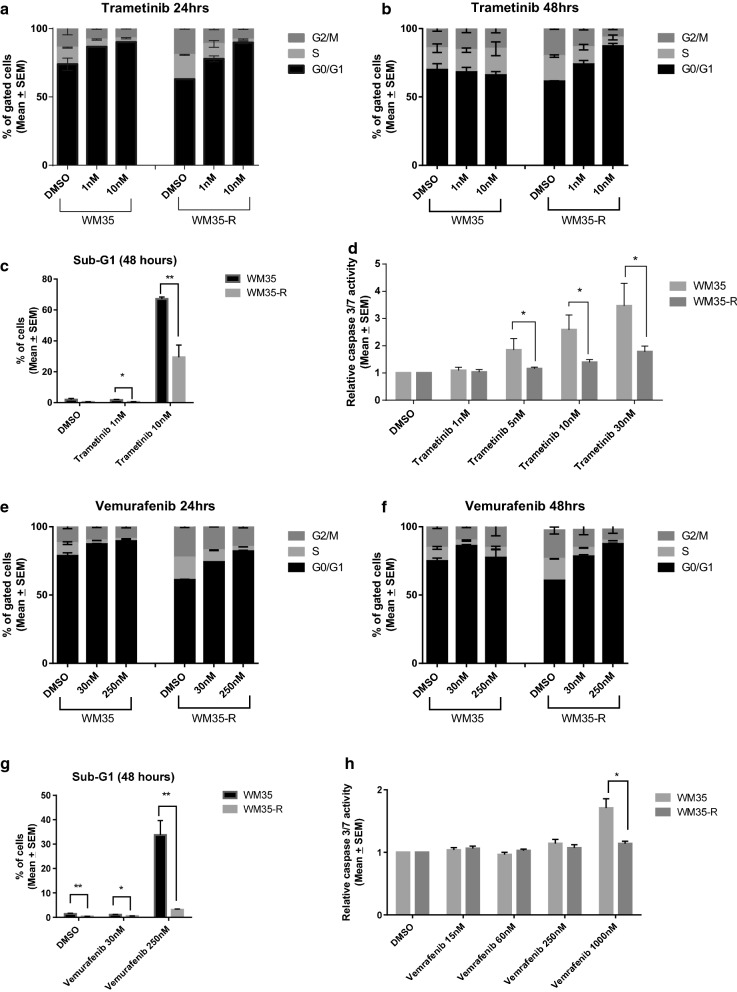
The effect of trametinib or vemurafenib treatment on cell cycle distribution and apoptosis for paired WM35 and WM35-R. Cell cycle distribution changes for 24 (**a**, **e**) and 48 (**b**, **f**) hour treatment with trametinib (**a**, **b**) or vemurafenib (**e**, **f**) by FACS analysis. Sub-G1 events by FACS (**c**, **g**) and caspase 3/7 activity (**d**, **h**), were used as indicators of apoptosis. Statistically significant p-values (*p < 0.05) are indicated. Data are presented as mean ± standard error of mean (SEM) for at least three independent repeats

To further explore the different responses of WM35 and WM35-R, FACS analysis was used to evaluate the cell cycle distribution after MAPK inhibitor treatments. Both trametinib and vemurafenib induced dose dependent G1 arrest in WM35 and WM35-R after 24-h (Fig. 4a, e, Additional file 1: Figure S3A, C, S4A, C). After 48-h treatments, continued G1 arrest was evident for WM35-R, however for WM35 the proportion of cells in G1 phase was not different to DMSO control, but there was an increase in sub-G1 signals (Fig. 4b, c, f, g, Additional file 1: Figures S3B, D, S4B, D). Both trametinib and vemurafenib induced dose dependent sub-G1 signals indicative of apoptosis in WM35 after 48-h treatment, which were significantly reduced in WM35-R (Fig. 4c, g). Caspase 3/7 activity measurements supported the findings that trametinib and vemurafenib induced more apoptosis in WM35 than WM35-R (Fig. 4d, h).

### WM35-R showed much lower induction of p53-dependent gene transcripts than WM35 after 24 h trametinib treatment

**Fig. 5:**
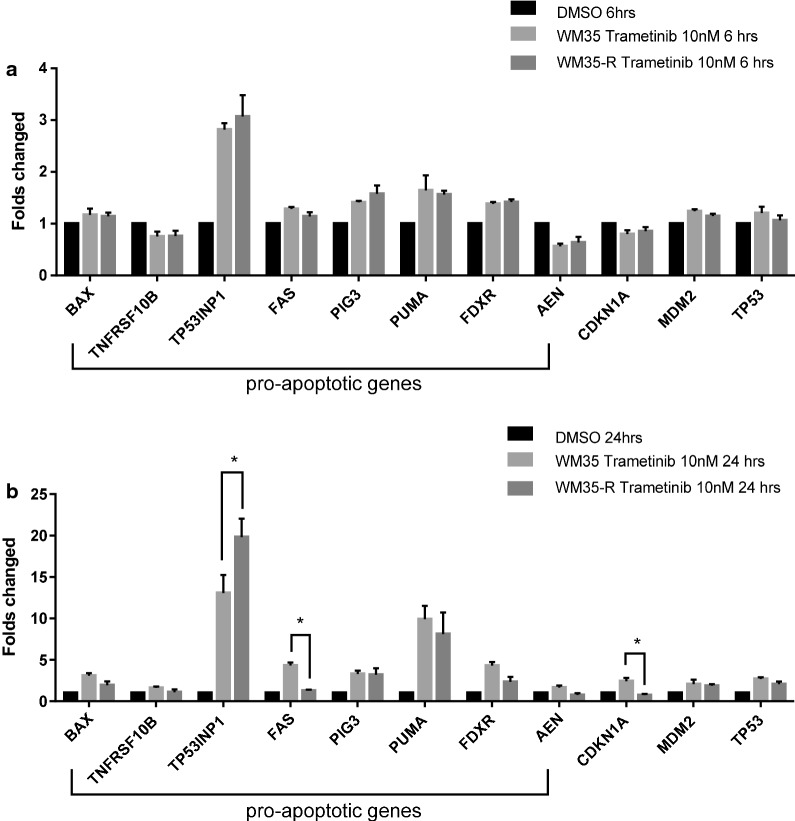
Effect of trametinib on mRNA expression of p53 transcriptional target genes by qRT-PCR. mRNA expression of p53 transcriptionally regulated genes in response to 10 nM tramentinb for 6 (**a**) and 24 (**b**) hours relative to DMSO solvent and GAPDH control in WM35 and WM35-R. Statistically significant differences (*p < 0.05) between WM35 and WM35-R are shown above the bars for trametinib treatment normalised to DMSO control. Data are presented as mean ± standard error of mean (SEM) for three independent repeats

Markedly different apoptotic responses were observed for WM35 compared with WM35-R following treatment with the MAPK pathway inhibitors. This suggested the hypothesis that WM35-R with a non-functional p53 was deficient for the induction of p53-dependent apoptosis in response to trametinib or vemurafenib. To investigate this further, a panel of p53 transcriptional target genes was examined by qRT-PCR (Fig. 5). WM35 and WM35-R were treated with 10 nM trametinib, a dose which had generated significantly different apoptotic responses. All the pro-apoptotic genes except *TP53INP1* were induced to a lower extent in WM35-R than WM35 after 24-h trametinib treatment. Interestingly, the *TP53* transcripts slightly increased after trametinib treatment, suggesting MAPK activity inhibited *TP53* transcription, which was reversed by trametinib in WM35. The lack of p53 transcriptional target gene induction in the p53^MUT^ WM35-R cells was consistent with the functional inactivation of p53 and indicated that the parental WM35 apoptotic response to trametinib and vemurafenib was at least partly dependent on p53. However, the growth inhibitory response to the MAPK pathway inhibitors at lower doses and earlier time points was less dependent on p53. Only a modest increase of *CDKN1A* was found in WM35 but not in WM35-R.

### SiRNA-mediated knockdown of p53 reduced sensitivity and caspase 3/7 activity after trametinib and vemurafenib

**Fig. 6:**
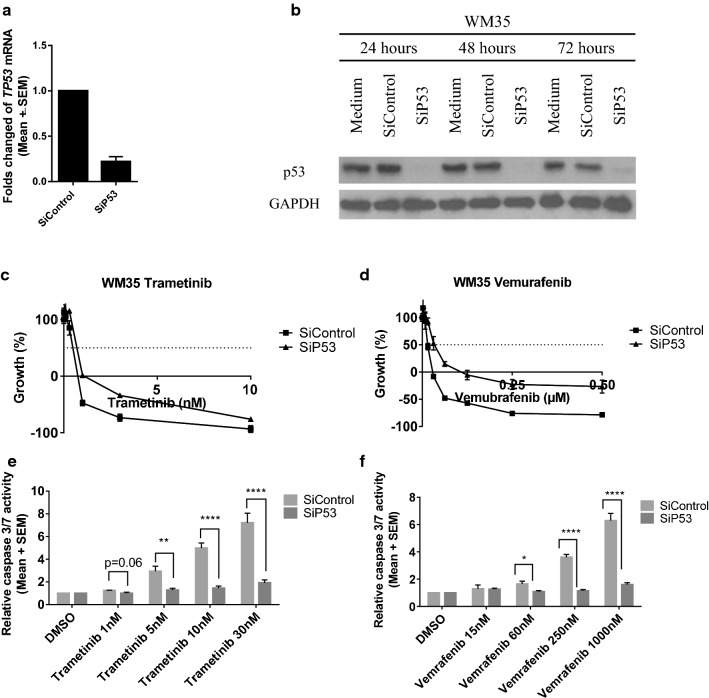
SiRNA mediated knockdown of p53 in WM35 reduced apoptosis after trametinib or vemurafenib treatment. **a** mRNA expression of p53 after 24 h siRNA mediated knockdown, relative to SiControl and GAPDH control in WM35. **b** p53 protein levels after siRNA mediated knockdown for 24, 48 and 72 h detected by immumoblotting. SRB assay of growth inhibition for WM35 after 24-h siRNA treatment followed by 72-h trametinib (**c**) or vemurafenib (**d**) treatments. **e**, **f** Caspase 3/7 activity in WM35 after 24-h siRNA treatment followed by 24-h trametinib (**c**) or vemurafenib (**d**) treatments. Statistically significant differences (*p < 0.05, **p < 0.01, ***p < 0.001, ****p < 0.0001) between SiRNA p53 knockdown and SiRNA control treated cells are shown above the bars for each treatment normalised to DMSO control. Data are presented as mean ± standard error of mean (SEM) for three independent repeats

To test the hypothesis that p53 is responsible for trametinib- and vemurafenib-induced apoptosis, p53 levels were suppressed by siRNA in WM35 and A375. Immunoblotting and qRT-PCR confirmed the efficiency of p53 knockdown by siRNA (Fig. 6a, b, Additional file 1: Figure S5A, B). After siRNA-mediated knockdown of p53, cells were treated with trametinib and vemurafenib. Compared to siControl, a decrease in sensitivity to trametinib and vemurafenib was noted for both A375 and WM35 after siRNA-mediated knockdown of p53 (Fig. 6c, d, and Additional file 1: Figure S5C, D). Significant suppression of caspase 3/7 activities was found in both cells after knockdown of p53 confirming that p53 was at least partially responsible for trametinib- and vemurafenib-induced apoptosis (Fig. 6e, f, Additional file 1: Figure S5E, F).

### The response of the paired SJSA-1 and SN40R2 cell lines to trametinib

**Fig. 7:**
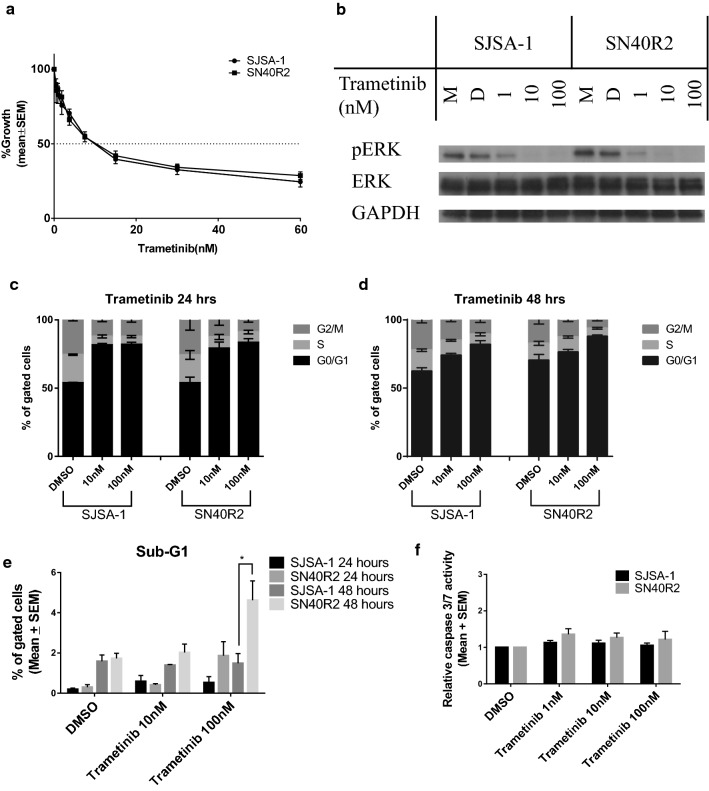
The effect of trametinib treatment on paired SJSA-1 and SN40R2. **a** Growth inhibition curves by SRB assay of SJSA-1 and SN40R2 treated with trametinib for 72 h. **b** Immunobloting of SJSA-1 and SN40R2 after trametinib for 6 h. Cell cycle distribution changes for 24 (**c**) and 48 (**d**) hour treatment with trametinib measured by FACS analysis. Sub-G1 events by FACS (**e**) and caspase 3/7 activity (**f**) after 24 h treatment, as indicators of apoptosis. Statistically significant p-values (*p < 0.05) are indicated where appropriate. Data are presented as mean ± standard error of mean (SEM) for at least three independent repeats

SJSA-1 and SN40R2 cell lines have an *NRAS* mutation resulting in MAPK activation, suggesting possible response to trametinib. Growth inhibition assays showed both of the paired cell lines were sensitive to trametinib. The growth inhibition curves for both cell lines closely overlapped and did not go below the X axis (basal level). This suggested no or little apoptosis was induced after trametinib treatment in either cell line (Fig. 7a). Immunoblotting confirmed the on-target effect of trametinib, evidenced by suppression of phospho-ERK (Fig. 7b). FACS cell cycle distribution analysis showed a dose-dependent G1 arrest response to trametinib for both cell lines (Fig. 7c, d) and only a small Sub-G1 fraction component was noted after treatment for 24 and 48 h. Unexpectedly, the resistant SN40R2 cell showed more Sub-G1 signals after 100 nM trametinib treatment for 48 h than the parental SJSA-1 cells (Fig. 7e). However, there was little caspase 3/7 induction in both cell lines and although SN40R2 showed a slightly higher caspase 3/7 activity than SJSA-1, this was not statistically significant (Fig. 7f). The sub-G1 observation for the 100 nM response by FACS analysis was not mirrored by the caspase 3/7 activity following that dose of trametinib.

## Discussion

**Fig. 8:**
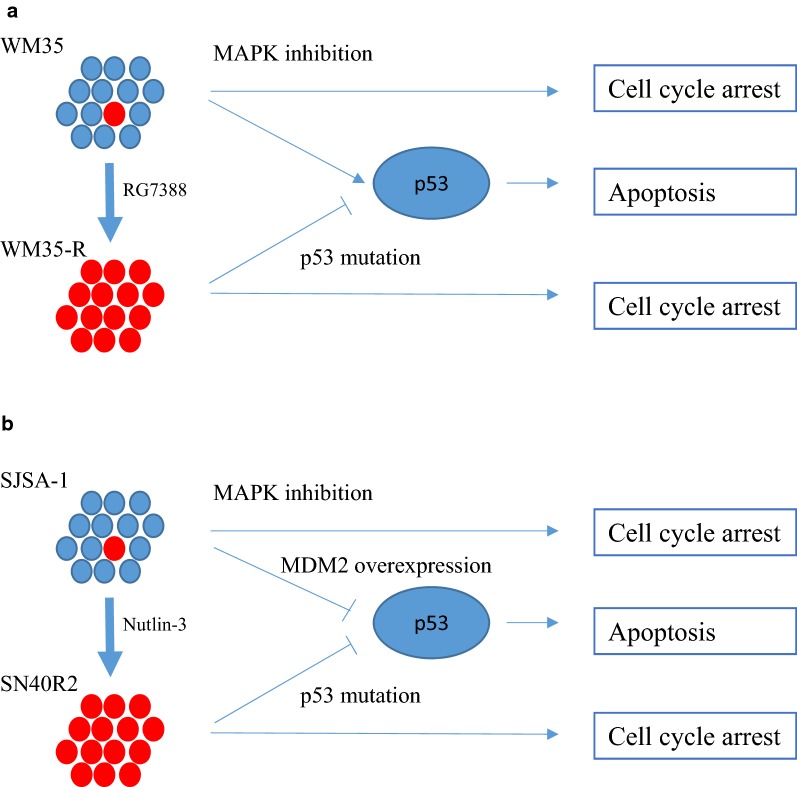
Proposed model for MAPK inhibition in paired *TP53*^WT^ and *TP53*^MUT^ cell lines selected for resistance to MDM2 inhibitors. WM35-R (**a**) and SN40R2 (**b**) were selected from WM35 and SJSA-1 respectively and both were sensitive to growth inhibition by MAPK pathway inhibition. The *TP53*^MUT^ cells lost p53-dependent apoptosis but retained p53-independent cell cycle arrest and growth inhibition in response to inhibition of the MAPK pathway. SJSA-1 showed minimal p53-dependent apoptosis in response to MAPK pathway inhibition, plausibly resulting from p53 suppression by overexpression of MDM2

In the current study, the growth inhibitory and cytotoxic activities of MAPK inhibitors, trametinib and vemurafenib, were investigated with paired *TP53*^*WT*^ and *TP53*^*MUT*^ cell lines. The results demonstrated that *TP53* mutant cells selected for resistance to MDM2 inhibitors nevertheless retained sensitivity to targeted treatment with MAPK inhibitors. Trametinib and vemurafenib showed similar growth inhibitory effects on paired WM35/WM35-R and SJSA-1/SN40R2 cells. However, WM35 underwent p53-dependent apoptosis after trametinib and vemurafenib treatment, whereas SJSA-1, which is *MDM2* amplified, did not undergo a p53-dependent apoptotic response to trametinib, although growth inhibition was observed. A diagrammatic summary is shown in Fig. 8.

### Cell lines selected for resistance to MDM2 inhibitors had *TP53* mutations and were cross-resistant to other MDM2 inhibitors

Most in vitro studies reported have shown that cells selected for resistance to MDM2 inhibitors harbour p53 mutations and show a deficiency of p53-dependent apoptosis in response to MDM2 inhibitor treatment [19, 20, 23]. One recent study used piggyBac transposon insertional mutagenesis in a cohort of allografts with an underlying *CDKN2A* deletion, to anticipate resistance mechanisms which might occur during treatment with the MDM2-p53 inhibitor HDM201. The most frequent mechanisms conferring resistance converged on direct (*TP53* mutations) or indirect (gain-of-function of *MDM4*, *TP63*, *TP73*) loss-of-function inactivation of the p53 protein. In addition, activation of the anti-apoptotic B-cell lymphoma-extra large (*Bcl*-*xL*) gene was observed [24]. Although MDM2 inhibitors (Nutlin-3, RG7388) were previously reported as a modulators of MDR-1(*p*-glycoprotein) [25, 26] and MDR-1-overexpressed cells showed decreased sensitivity to Nutlin-3 [25], there are no reports that selection for resistance to MDM2 inhibitors results from amplification and/or overexpression of genes encoding multidrug resistance proteins.

Consistent with previous reports, both cell lines selected for resistance to MDM2 inhibitors investigated in the current study have *TP53* mutations. The selection and characterisation of SN40R2, which has been reported previously by our group [20] and of WM35-R described here, are the first reported p53^MUT^ cell lines which have been selected in vitro for resistance to RG7388 [22, 27], a clinically relevant more potent and specific MDM2 inhibitor than Nutlin-3 [19, 20, 23] and MI-63 [20] which were used in previous studies. In contrast to another study which was based on p53 mutation which had been generated de novo [27], the current study investigated the selection of cells resistant to MDM2 inhibitors from a parental culture of WM35 melanoma cells. This resulted in the selection of a p53 mutated subpopulation. Most reported TP53 mutations are located in the DNA binding domain, however the WM35-R resistant sub-line has an uncommonly reported p53^G334V^ point missense mutation in a critical residue the tetramerization domain of p53, which consists of a α-strand (Glu326–Arg333), a tight turn (Gly334), and a β-helix (Arg335–Gly356) [28]. In the wild-type tetramerization domain, Gly334 facilitates the formation of a sharp turn connecting the β-strand with the α-helix and adopts a backbone conformation that would be energetically unfavorable for a non-glycine residue, indicating that mutation of Gly334 is expected to result in structural distortions [29]. The Gly334 mutant p53 protein exhibits a global decrease in DNA binding and transactivation activity [30]. In the current study, WM35-R with such a p53^G334V^ mutation lost its transactivation activity, evidenced by no induction of downstream targets of p53 and consequent cross-resistance to other MDM2 inhibitors. For the other resistant cell line, SN40R2, detailed characteristics and discussions have been addressed in our previous publication [20].

### *TP53*^MUT^ cells selected for resistance to MDM2 inhibitors retain sensitivity to growth inhibition by MAPK pathway inhibitors but a reduced p53-dependent apoptotic response to them

To investigate whether cross-resistance occurs between MDM2/p53 binding antagonists and MAPK pathway inhibitors, trametinib and vemurafenib, paired WM35/WM35-R and SJSA-1/SN40R2 cells were tested and showed similar growth inhibitory effects for the paired cell lines. This showed that a growth inhibitory response to MAPK pathway inhibition was maintained in the *TP53* mutant cell lines selected for resistance to MDM2 inhibitors. However, more p53-regulated apoptosis was found in WM35 than WM35-R, supported by sub-G1 fraction on FACS analysis and caspase 3/7 activity, indicating that trametinib and vemurafenib acted on the cells partially through a p53-regulated pro-apoptotic pathway, particularly at higher doses. The interaction between the MAPK pathways and the p53/MDM2/MDMX network are tightly regulated [31]. A murine model study showed that the RAS/RAF pathway activated the transcription of both MDM2 and its inhibitor p19^ARF^ [32]. The level of p53 was determined by opposing effects of RAF-induced p19^ARF^ and MDM2. In the absence of p19^ARF^ or when the induction of MDM2 exceeds that of p19^ARF^ (a common situation in many human cancers), the RAS/RAF/MEK/ERK pathway attenuates p53. Another study supported this finding by using a RAS inhibitor, farnesylthiosalicylic acid (FTS), which increased p53 expression through both downregulation of MDM2 and transcriptional activation of p53 [33]. Consistent with these observations, in the current study trametinib treatment induced transcripts of *TP53* as well as *CDKN1A* and p53-regulated pro-apoptotic genes in WM35. *CDKN1A* only slightly increased in WM35 but not in WM35-R indicating that trametinib-induced G1 arrest was mainly p53/p21-independent, which is consistent with a previous report that MEK inhibition in melanoma cells resulted in p27 regulated G1 arrest rather than via p21 [34].

### Differences between WM35 and SJSA-1

In contrast to the different responses between WM35 and WM35-R, the SJSA and SN40R2 cell lines did not show such differences. Little apoptosis was found in the parental *TP53* wild-type SJSA-1 cells, which was different to WM35. SJSA-1 is a *MDM2* amplified osteosarcoma cell line in which the function of p53 is suppressed by overexpressed MDM2, whereas WM35 has no MDM2 amplification and lower expression of MDM2 protein (Additional file 1: Figure S6). This possibly explains why little p53-dependent apoptosis was found after trametinib treatment of SJSA-1 and SN40R2. The MDM2 suppression of p53 rather than RAS mutation is a main driver target in SJSA-1, evidenced by the significant induction of caspase 3/7 activity after MDM2 inhibitor treatment (Additional file 1: Figure S7). The combination of MAPK and MDM2 inhibitors as a strategy for cancers with wild-type p53 and mutations of RAS/RAF merits further exploration.

## Conclusions

In conclusion, there was restricted cross-resistance between MDM2/p53 binding antagonists and MAPK inhibitors indicating that resistant cancer cells potentially selected by MDM2 inhibitors can nevertheless be treated with MAPK inhibitors if the cells have druggable mutations driving the MAPK pathway.

## Additional file

**Additional file 1.** Additional figures.

## Abbreviations

DMSO: dimethyl sulfoxide
DNA: deoxyribonucleic acid
FACS: fluorescence-activated cell sorting
MDEM: Dulbecco’s modified Eagle’s medium
MDR-1: multidrug resistance protein 1
MRP-1: multidrug-resistance-associated protein 1
MUT: mutant
PGP: *p*-glycoprotein
SEM: standard error of mean
siP53: SiRNA of p53
siControl: siRNA for control
SRB: sulforhodamine B
STR: serial tandem repeat
WT: wild type
qRT-PCR: quantitative real-time polymerase chain reaction

## References

[1] Prior IA, Lewis PD, Mattos C (2012). A comprehensive survey of Ras mutations in cancer. Cancer Res.

[2] Holderfield M, Deuker MM, McCormick F, McMahon M (2014). Targeting RAF kinases for cancer therapy: BRAF-mutated melanoma and beyond. Nat Rev Cancer.

[3] Larkin J, Ascierto PA, Dreno B, Atkinson V, Liszkay G, Maio M, Mandala M, Demidov L, Stroyakovskiy D, Thomas L (2014). Combined vemurafenib and cobimetinib in BRAF-mutated melanoma. N Engl J Med.

[4] Robert C, Karaszewska B, Schachter J, Rutkowski P, Mackiewicz A, Stroiakovski D, Lichinitser M, Dummer R, Grange F, Mortier L (2015). Improved overall survival in melanoma with combined dabrafenib and trametinib. N Engl J Med.

[5] Bollag G, Tsai J, Zhang J, Zhang C, Ibrahim P, Nolop K, Hirth P (2012). Vemurafenib: the first drug approved for BRAF-mutant cancer. Nat Rev Drug Discov.

[6] Wright CJ, McCormack PL (2013). Trametinib: first global approval. Drugs.

[7] Yamaguchi T, Kakefuda R, Tajima N, Sowa Y, Sakai T (2011). Antitumor activities of JTP-74057 (GSK1120212), a novel MEK1/2 inhibitor, on colorectal cancer cell lines in vitro and in vivo. Int J Oncol.

[8] Gilmartin AG, Bleam MR, Groy A, Moss KG, Minthorn EA, Kulkarni SG, Rominger CM, Erskine S, Fisher KE, Yang J (2011). GSK1120212 (JTP-74057) is an inhibitor of MEK activity and activation with favorable pharmacokinetic properties for sustained in vivo pathway inhibition. Clin Cancer Res.

[9] Wade M, Wang YV, Wahl GM (2010). The p53 orchestra: Mdm2 and Mdmx set the tone. Trends Cell Biol.

[10] Levine AJ, Momand J, Finlay CA (1991). The p53 tumour suppressor gene. Nature.

[11] Fischer M (2017). Census and evaluation of p53 target genes. Oncogene.

[12] Muller PA, Vousden KH (2013). p53 mutations in cancer. Nat Cell Biol.

[13] Brown CJ, Lain S, Verma CS, Fersht AR, Lane DP (2009). Awakening guardian angels: drugging the p53 pathway. Nat Rev Cancer.

[14] Vassilev LT, Vu BT, Graves B, Carvajal D, Podlaski F, Filipovic Z, Kong N, Kammlott U, Lukacs C, Klein C (2004). In vivo activation of the p53 pathway by small-molecule antagonists of MDM2. Science.

[15] Ding Q, Zhang Z, Liu JJ, Jiang N, Zhang J, Ross TM, Chu XJ, Bartkovitz D, Podlaski F, Janson C (2013). Discovery of RG7388, a potent and selective p53-MDM2 inhibitor in clinical development. J Med Chem.

[16] Hyman D, Chatterjee M, Langenberg MHG, Lin CC, Suarez C, Tai D, Cassier P, Yamamoto N, De Weger VA, Jeay S (2016). Dose- and regimen-finding phase I study of NVP-HDM201 in patients (pts) with TP53 wild-type (wt) advanced tumors. Eur J Cancer.

[17] Gottesman MM, Fojo T, Bates SE (2002). Multidrug resistance in cancer: role of ATP-dependent transporters. Nat Rev Cancer.

[18] Chen DH, Zhang XS (2015). Targeted therapy: resistance and re-sensitization. Chin J Cancer.

[19] Michaelis M, Rothweiler F, Barth S, Cinatl J, van Rikxoort M, Loschmann N, Voges Y, Breitling R, von Deimling A, Rodel F (2011). Adaptation of cancer cells from different entities to the MDM2 inhibitor Nutlin-3 results in the emergence of p53-mutated multi-drug-resistant cancer cells. Cell Death Dis.

[20] Drummond CJ, Esfandiari A, Liu J, Lu X, Hutton C, Jackson J, Bennaceur K, Xu Q, Makimanejavali AR, Del Bello F (2016). TP53 mutant MDM2-amplified cell lines selected for resistance to MDM2-p53 binding antagonists retain sensitivity to ionizing radiation. Oncotarget.

[21] Skehan P, Storeng R, Scudiero D, Monks A, McMahon J, Vistica D, Warren JT, Bokesch H, Kenney S, Boyd MR (1990). New colorimetric cytotoxicity assay for anticancer-drug screening. J Natl Cancer Inst.

[22] Wu CE, Esfandiari A, Ho YH, Wang N, Mahdi AK, Aptullahoglu E, Lovat P, Lunec J (2018). Targeting negative regulation of p53 by MDM2 and WIP1 as a therapeutic strategy in cutaneous melanoma. Br J Cancer.

[23] Aziz MH, Shen H, Maki CG (2011). Acquisition of p53 mutations in response to the non-genotoxic p53 activator Nutlin-3. Oncogene.

[24] Chapeau EA, Gembarska A, Durand EY, Mandon E, Estadieu C, Romanet V, Wiesmann M, Tiedt R, Lehar J, de Weck A (2017). Resistance mechanisms to TP53-MDM2 inhibition identified by in vivo piggyBac transposon mutagenesis screen in an Arf−/− mouse model. Proc Natl Acad Sci USA.

[25] Chen L, Zhao Y, Halliday GC, Berry P, Rousseau RF, Middleton SA, Nichols GL, Del Bello F, Piergentili A, Newell DR (2014). Structurally diverse MDM2-p53 antagonists act as modulators of MDR-1 function in neuroblastoma. Br J Cancer.

[26] Michaelis M, Rothweiler F, Klassert D, von Deimling A, Weber K, Fehse B, Kammerer B, Doerr HW, Cinatl J (2009). Reversal of *P*-glycoprotein-mediated multidrug resistance by the murine double minute 2 antagonist Nutlin-3. Can Res.

[27] Skalniak L, Kocik J, Polak J, Skalniak A, Rak M, Wolnicka-Glubisz A, Holak TA (2018). Prolonged idasanutlin (RG7388) treatment leads to the generation of p53-mutated cells. Cancers (Basel).

[28] Chene P (2001). The role of tetramerization in p53 function. Oncogene.

[29] Joerger AC, Fersht AR (2007). Structure–function–rescue: the diverse nature of common p53 cancer mutants. Oncogene.

[30] Kamada R, Nomura T, Anderson CW, Sakaguchi K (2011). Cancer-associated p53 tetramerization domain mutants: quantitative analysis reveals a low threshold for tumor suppressor inactivation. J Biol Chem.

[31] Wu GS (2004). The functional interactions between the p53 and MAPK signaling pathways. Cancer Biol Ther.

[32] Ries S, Biederer C, Woods D, Shifman O, Shirasawa S, Sasazuki T, McMahon M, Oren M, McCormick F (2000). Opposing effects of Ras on p53: transcriptional activation of mdm2 and induction of p19ARF. Cell.

[33] Halaschek-Wiener J, Wacheck V, Kloog Y, Jansen B (2004). Ras inhibition leads to transcriptional activation of p53 and down-regulation of Mdm2: two mechanisms that cooperatively increase p53 function in colon cancer cells. Cell Signal.

[34] Kortylewski M, Heinrich PC, Kauffmann ME, Bohm M, MacKiewicz A, Behrmann I (2001). Mitogen-activated protein kinases control p27/Kip1 expression and growth of human melanoma cells. Biochem J.

