# Supplementary material for: TP53 mutant cell lines selected for resistance to MDM2 inhibitors retain growth inhibition by MAPK pathway inhibitors but a reduced apoptotic response
Source: Cancer Cell Int. 2019 Mar 7;19:53. doi: 10.1186/s12935-019-0768-3 (PMC6407233; doi:10.1186/s12935-019-0768-3)
Supplement: Supplementary file 1 — Additional file 1. Additional figures. [file 12935_2019_768_MOESM1_ESM.pdf]

A

STR profiling data

	Submitted sample profile				Reference database profile			
Loci	Sample profile ID:				Database profile ID:			
TH01	10				9.3			
TPOX	8				8			
vWA	17				17			
CSF1PO	10				10			
D16S539	12	13			12	13		
D7S820	10	13			10	13		
D13S317	11				11			
D5S818	11	12			11	12		
Amelogenin	X				X			
Multiple peaks detected? (indicating sample is a mixture)						No		
Alleles shared between sample and reference profiles						11		
Total alleles in submitted sample profile						12		
Total alleles in reference database profile						12		
Percent allelic match between sample and reference profiles						**91.67 %**		

B

STR profiling data

	Submitted sample profile				Reference database profile			
Loci	Sample profile ID:				Database profile ID:			
TH01	10				9.3			
TPOX	8				8			
vWA	17				17			
CSF1PO	10				10			
D16S539	12	13			12	13		
D7S820	10	13			10	13		
D13S317	11				11			
D5S818	11	12			11	12		
Amelogenin	X				X			
Multiple peaks detected? (indicating sample is a mixture)						No		
Alleles shared between sample and reference profiles						11		
Total alleles in submitted sample profile						12		
Total alleles in reference database profile						12		
Percent allelic match between sample and reference profiles						**91.67 %**		

Additional Figure S1a: Cell line authentication by Short tandem repeat (STR) profiling verified that the resistant WM35-R cells were derived from the WM35 parental cell line. WM35 and WM35-R cells had the same STR allele profile as the ATCC reference profile for WM35 cells (CRL-2807). A: WM35; B: WM35-R.

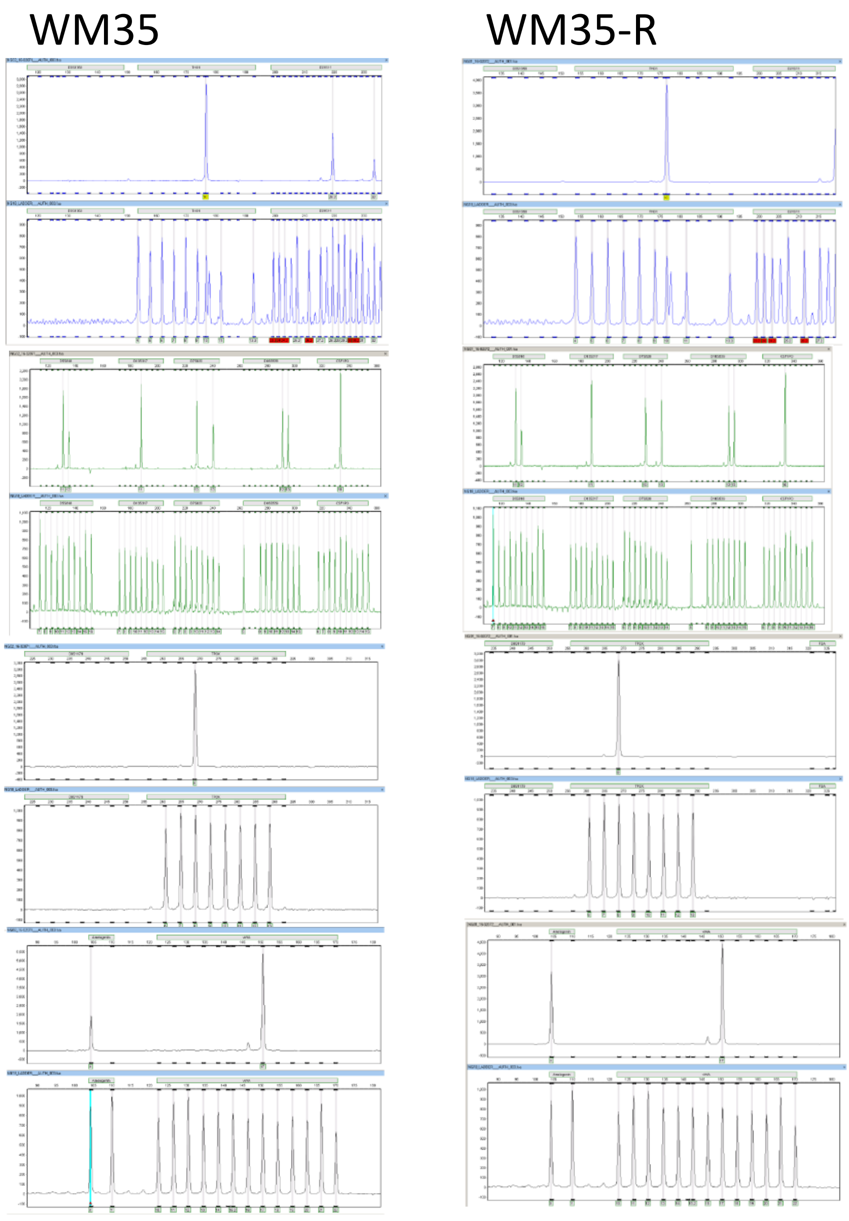

Additional Figure S1b: Cell line authentication showing close identity of the detailed short tandem repeat (STR) peak profiles between WM35 and WM35-R cells for a comparison of eight alleles.

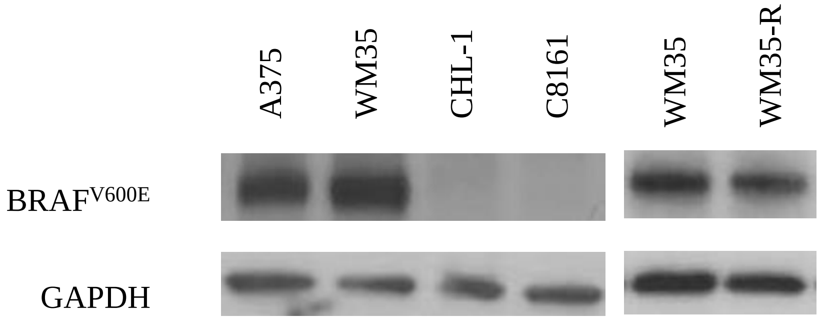

Additional Figure S2: Immunoblotting of BRAF^{V600E} with a mutation specific antibody (VE-1) in a panel of melanoma cell lines. A375, WM35 harbour *BRAF* (V600E) mutation and CHL-1, C8161 don’t. WM35-R retains BRAF^{V600E} expression.

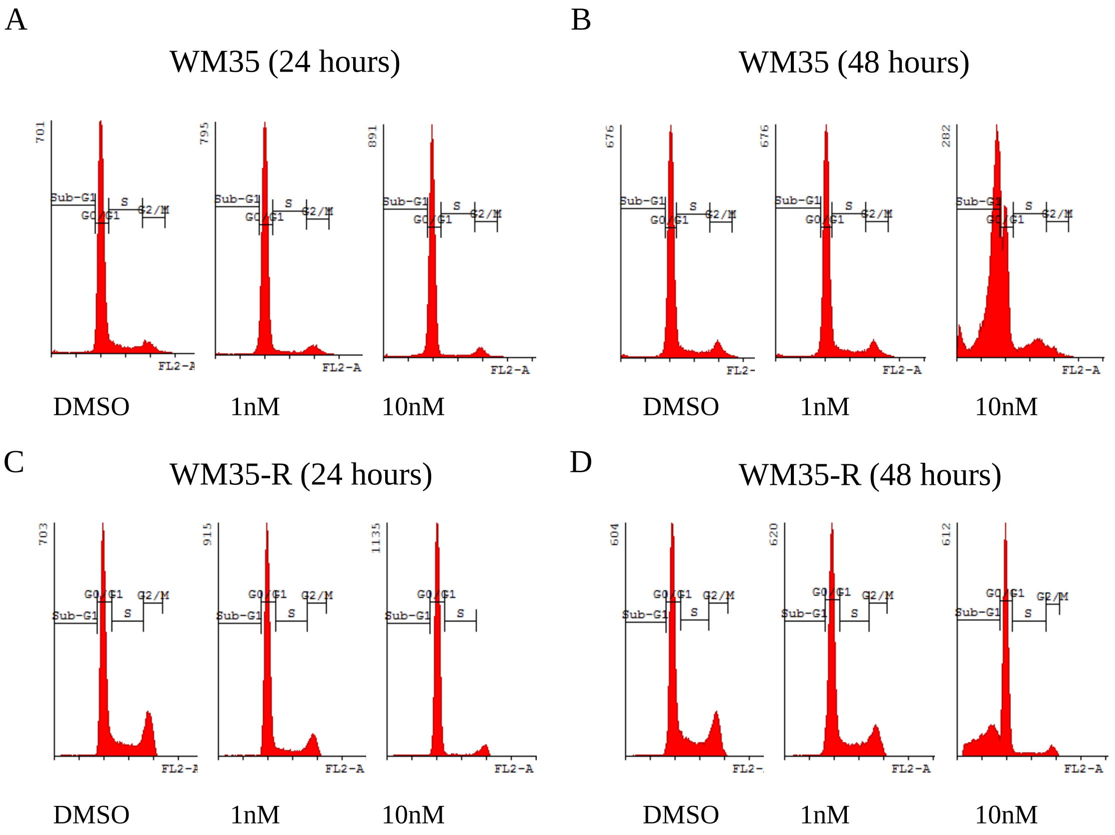

Additional Figure S3. FACS DNA content histograms for the cells treated with trametinib. WM35 and WM35-R were treated with trametinib at the indicated concentrations for 24 (A, C) and 48 (B, D) hours. WM35 (A, B) and WM35-R (C, D).

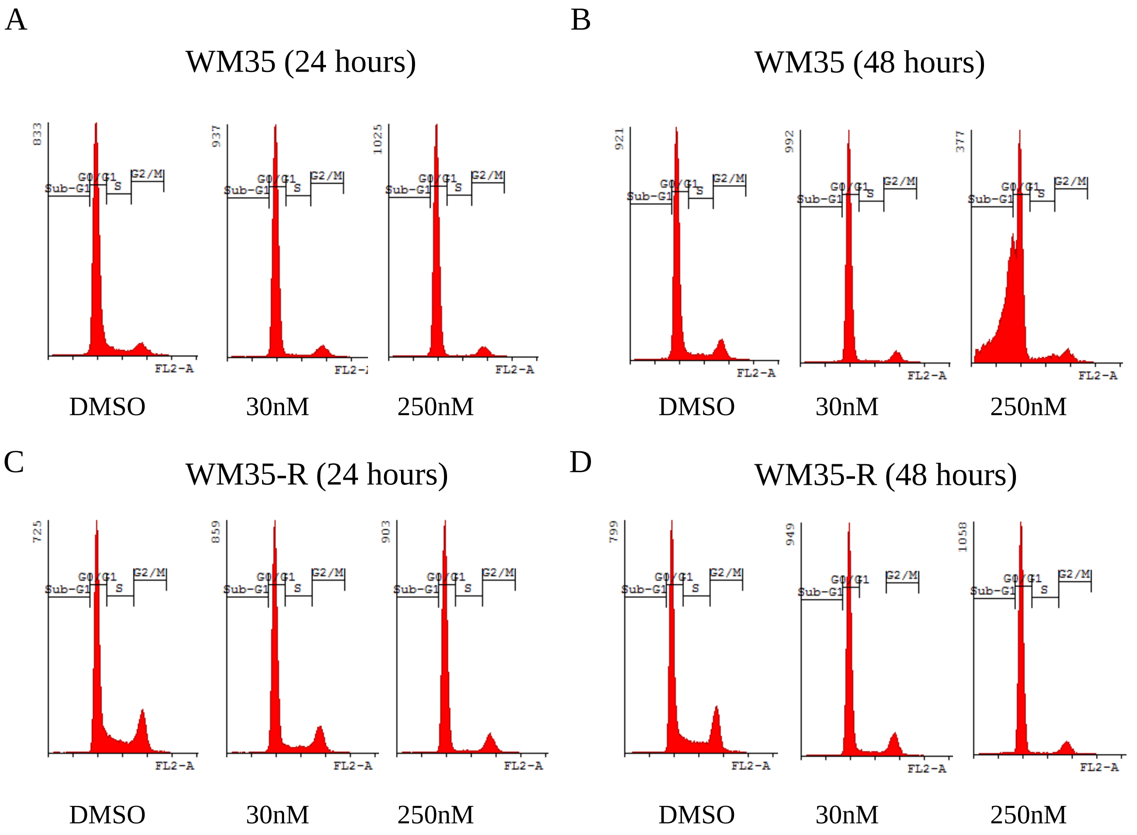

Additional Figure S4. FACS DNA content histograms for the cells treated with vemurafinib. WM35 and WM35-R were treated with vemurafenib at the indicated concentrations for 24 (A, C) and 48 (B, D) hours. WM35 (A, B) and WM35-R (C, D).

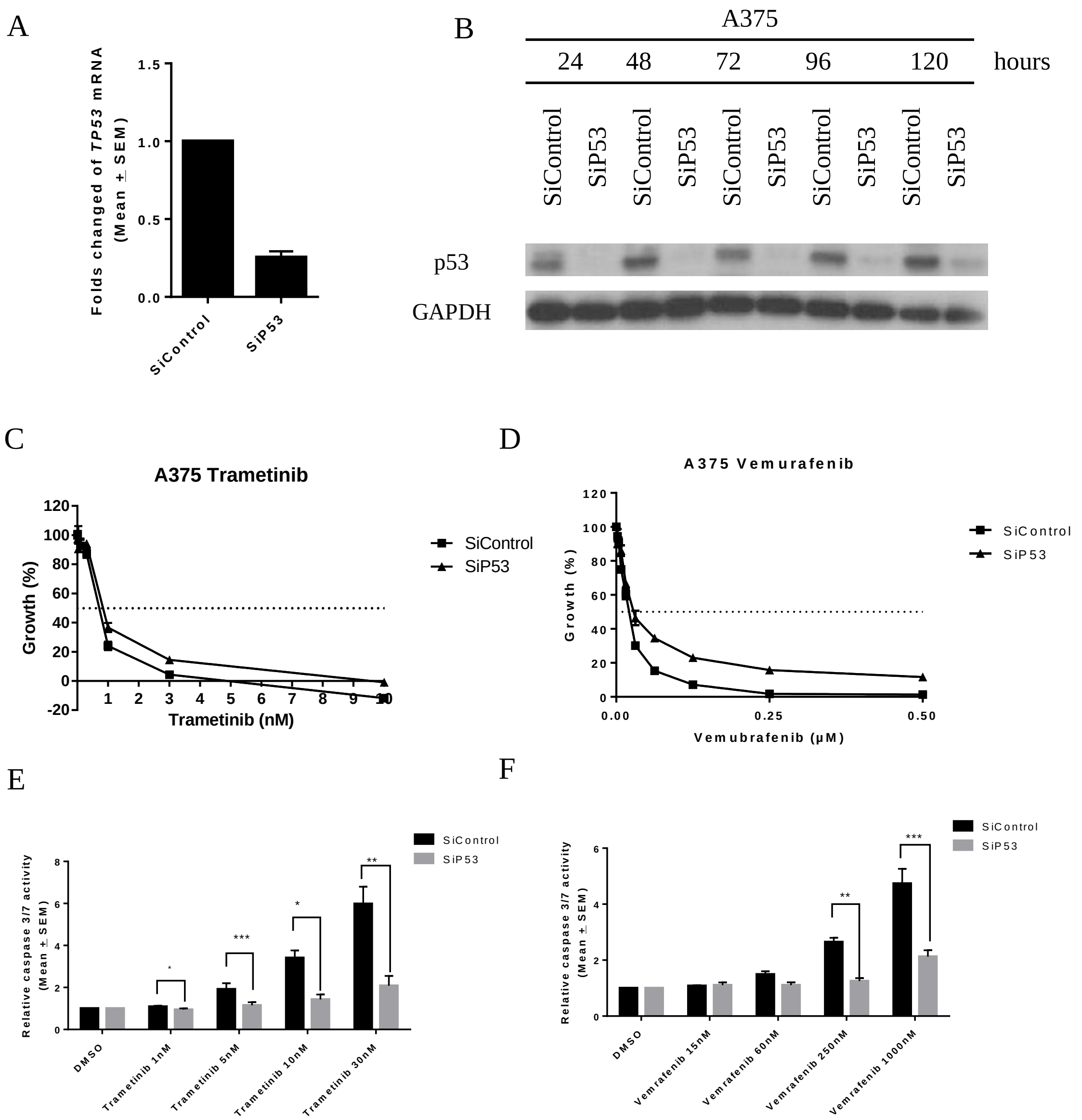

Additional Figure S5. **SiRNA mediated knockdown of p53 in A375 cells supressed apoptosis after trametinib or vemurafenib treatment.** (A) mRNA expression of p53 after 24 hour siRNA mediated knockdown, relative to SiControl and GAPDH control in A375. (B) p53 protein expression detected by immumoblotting after siRNA mediated knockdown for the indicated times. (C, D) SRB growth inhibition assay for A375 after 24-hour siRNA treatment followed by 72-hour trametimib (C) or vemurafenib (D) treatments. (E, F) Caspase 3/7 activity on WM35 after 24-hour siRNA treatment followed by 24-hour trametimib (E) or vemurafenib (F) treatments. Statistically significant differences (* $p < 0.05$, ** $p<0.01$, *** $p<0.001$, ****$p<0.0001$) resulting from siRNA p53 knockdown are shown above the bars for the effect of each treatment normalised to DMSO control. Data are presented as mean ± standard error of mean (SEM) for three independent repeats.

A

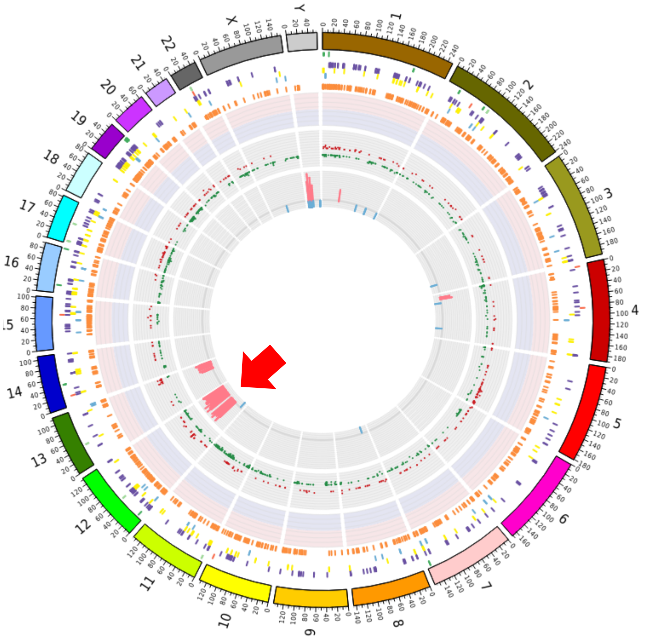

B

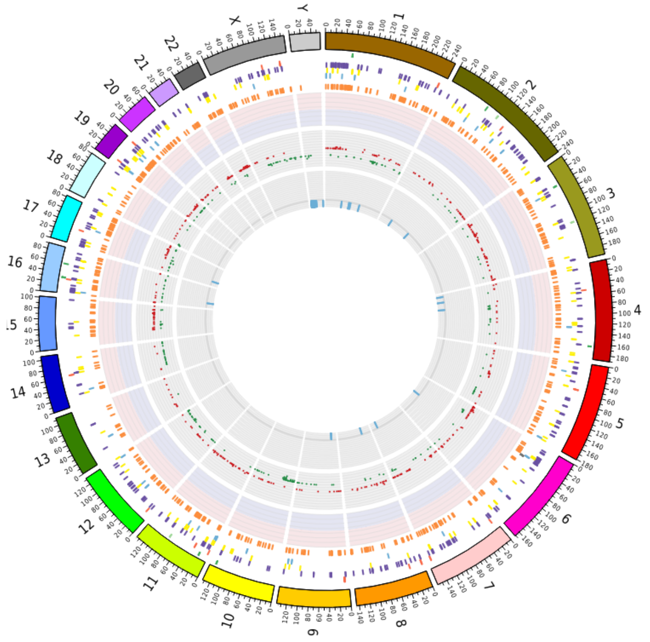

C

Sample	Expression	Expr Level (Z-Score)	CN Type	Minor Allele	Copy Number	CN Segment Position	Average Ploidy	Study	CNV
SJSA-1	Over	3.32	Gain	1	13	12:68753516..68899908	2.01	619	6571997

D

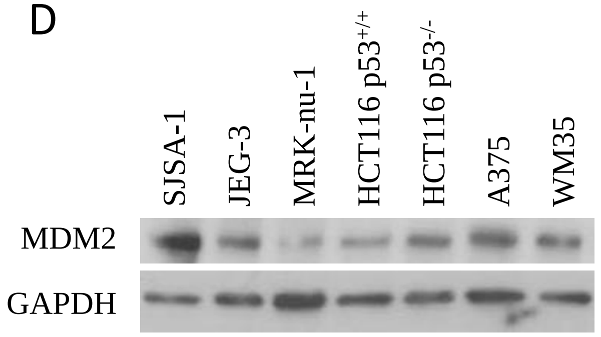

Additional Figure S6. MDM2 expression of WM35 and SJSA-1. (A,B) CIRCOS plots from COSMIC databank (assessed on 7th, Nov, 2018) show copy number gains on Chromosome 12q including the *MDM2* region in SJSA-1 (A, indicated by red arrow) and absence of copy number gains on chromosome 12q or any other chromosomal region of WM35 (B). (C) COSMIC data for SJSA-1 shows overexpression of *MDM2* mRNA and 13 copies of the gene (D) Immunoblotting showing higher basal expressions of MDM2 protein in a panel of cells including SJSA-1 and WM35.

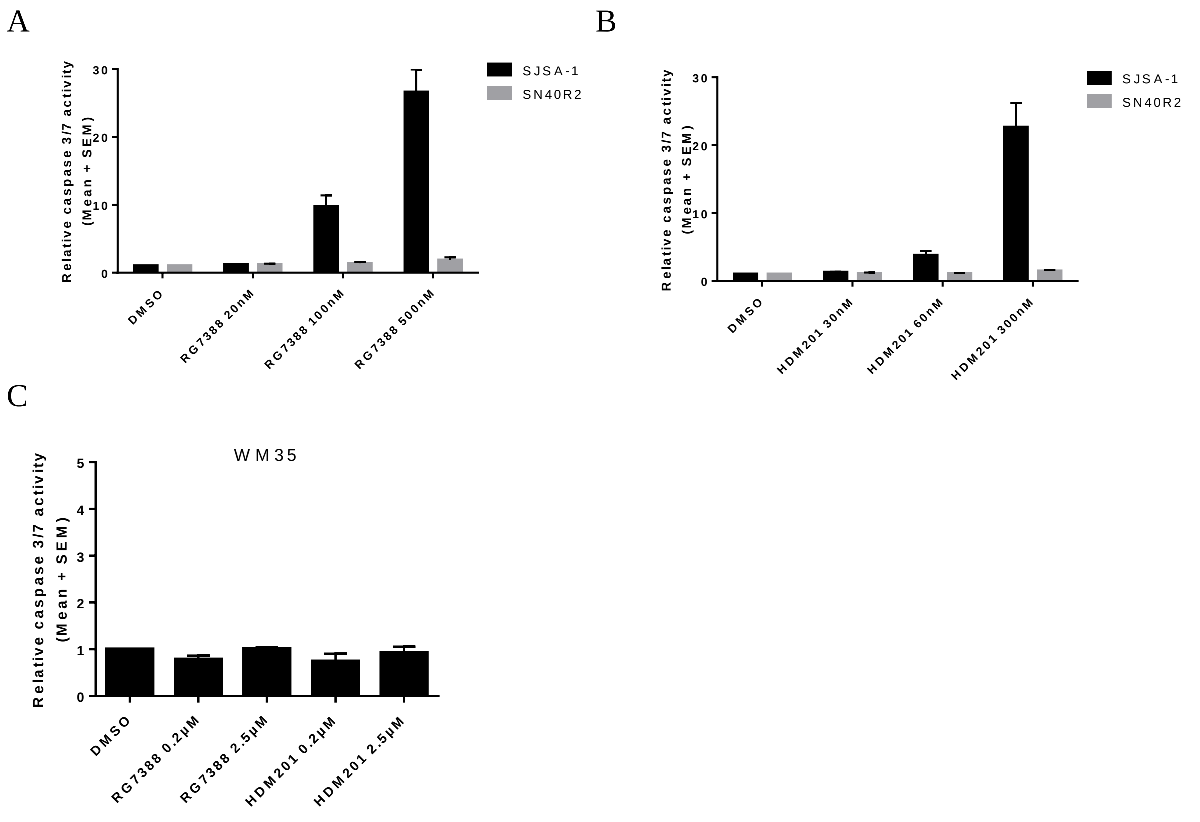

Additional Figure S7. **Caspase 3/7 activity in response to MDM2 inhibitor treatment.** Caspase 3/7 activities for SJSA-1 and SN40R following treatment with RG7388 (A) or HDM201 (B) for 24 hours. (C) Caspase 3/7 activity of WM35 following treatment with either RG7388 or HDM201.
